# Supplementary figures and images for: Cytotoxic alkyl-quinolones mediate surface-induced virulence in Pseudomonas aeruginosa
Source: PLoS Pathog. 2020 Sep 14;16(9):e1008867. doi: 10.1371/journal.ppat.1008867 (PMC7515202; doi:10.1371/journal.ppat.1008867)

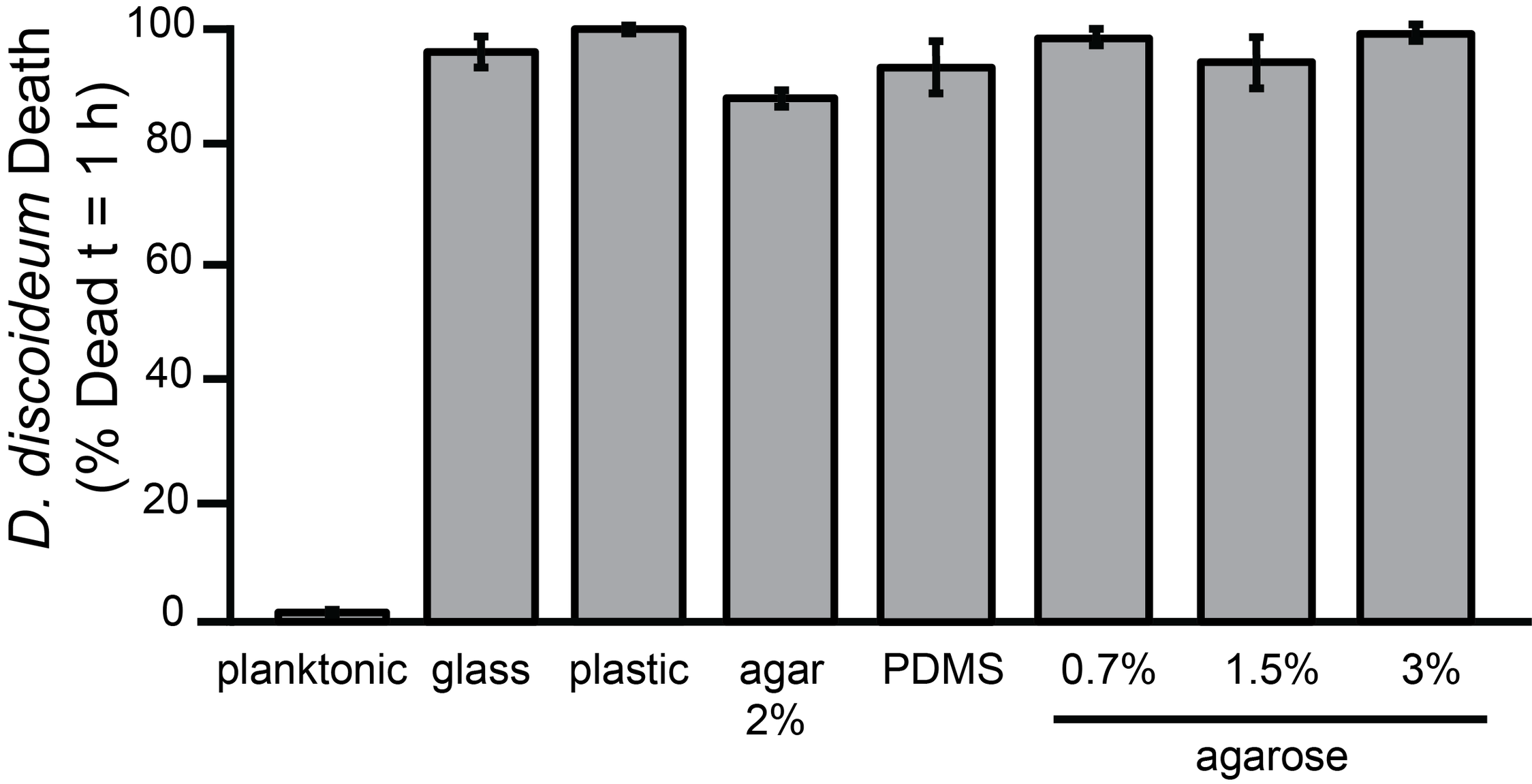

Supplement: S1 Fig — Quantification of D. discoideum killing by planktonic and surface-attached P. aeruginosa attached to substrates of various composition and stiffness after 1 h of co-culture. Cell death was indicated by positive staining by the fluorescent dye calcein-AM. Values are averages of three biological replicates and error bars represent standard error. Approximately 250–300 cells were analyzed for each measurement. PDMS is Sylgard-527. Values for the planktonic and glass condition are those in Fig 4B. (TIF) [file ppat.1008867.s003.tif]

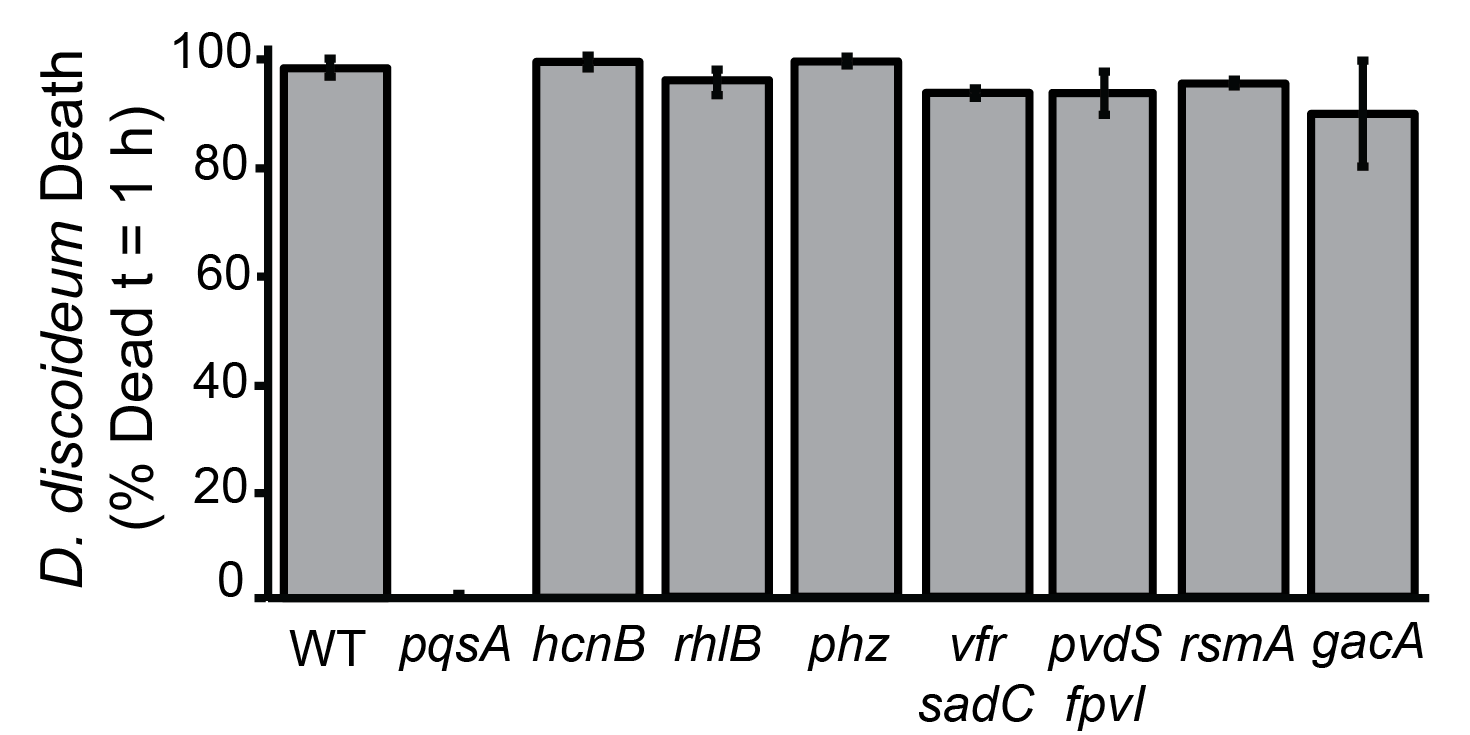

Supplement: S2 Fig — Quantification of D. discoideum killing by surface-attached P. aeruginosa mutants after 1 h of co-culture. Cell death was indicated by positive staining by the fluorescent dye calcein-AM. Values are averages of three independent experiments and error bars represent standard error. Approximately 150–300 cells were analyzed for each measurement. (TIF) [file ppat.1008867.s004.tif]

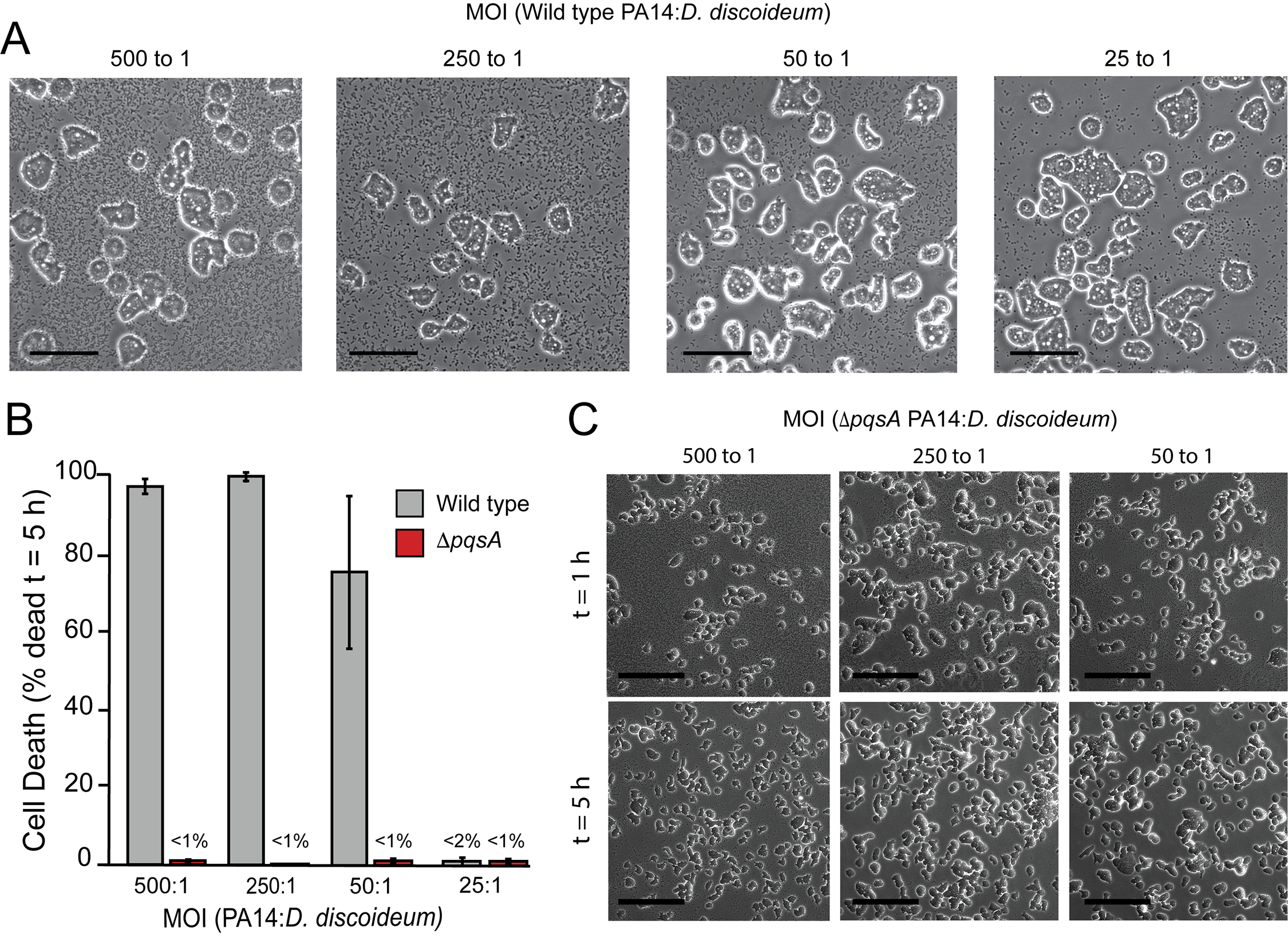

Supplement: S3 Fig — Quantification of D. discoideum killing by surface-attached wild type and pqsA mutant P. aeruginosa at varying initial MOI (P. aeruginosa to D. discoideum). (A) Representative images of P. aeruginosa mixed with D. discoideum at the labelled MOI at t = 0 h (scale bars = 50 μm). (B) Quantification of D. discoideum death after 5 h. Values are averages of three biological replicates and error bars represent standard error. Approximately 250–300 cells were analyzed for each measurement. Representative images of D. discoideum treated with pqsA mutant bacteria at varying MOI, demonstrating near-complete phagocytosis of bacterial lawns after 5 h when initial MOI is at or below 250:1 (scale bars = 150 μm). (TIF) [file ppat.1008867.s005.tif]

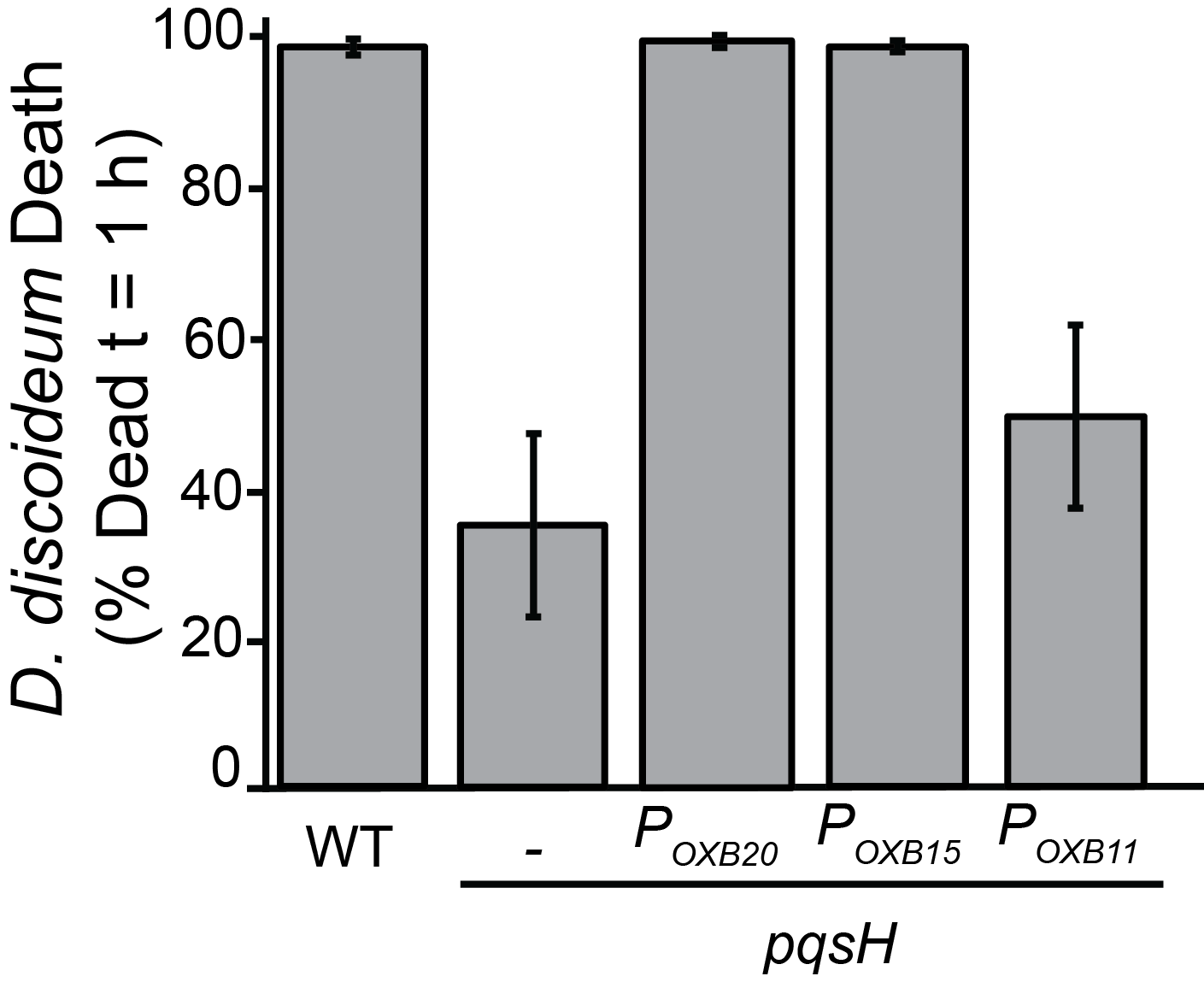

Supplement: S4 Fig — Quantification of D. discoideum killing by surface-attached P. aeruginosa pqsH mutants after 1 h of co-culture. Expression of pqsABCDE genes are controlled by a constitutive promoter with high (POXB20), moderate (POXB15), or low (POXB11) levels of expression. Cell death was indicated by positive staining by the fluorescent dye calcein-AM. Data for wild type and pqsH are from Fig 1C (n = 3). Other values shown are the average of two independent experiments and error bars represent standard deviation. Approximately 300–500 cells were analyzed for each measurement. (TIF) [file ppat.1008867.s006.tif]

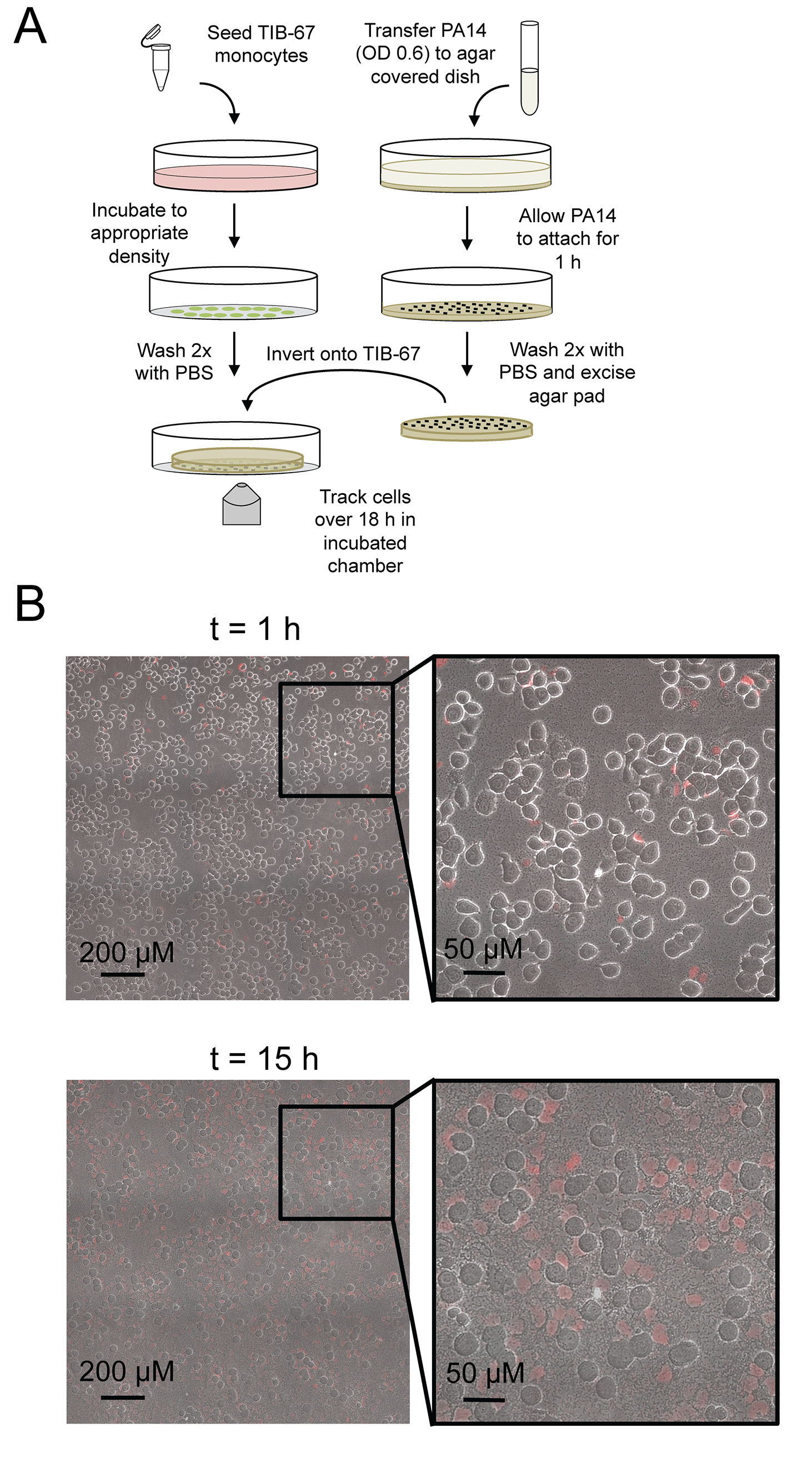

Supplement: S5 Fig — (A) Schematic of the monocyte cell-death assay described in Materials and Methods. (B) Representative images of TIB-67 monocytes treated with surface-attached wild type P. aeruginosa at a MOI of approximately 50:1 (bacteria:amoeba) after 1 and 15 h of incubation at 30°C. Cell death is indicated by propidium iodide staining. (TIF) [file ppat.1008867.s007.tif]

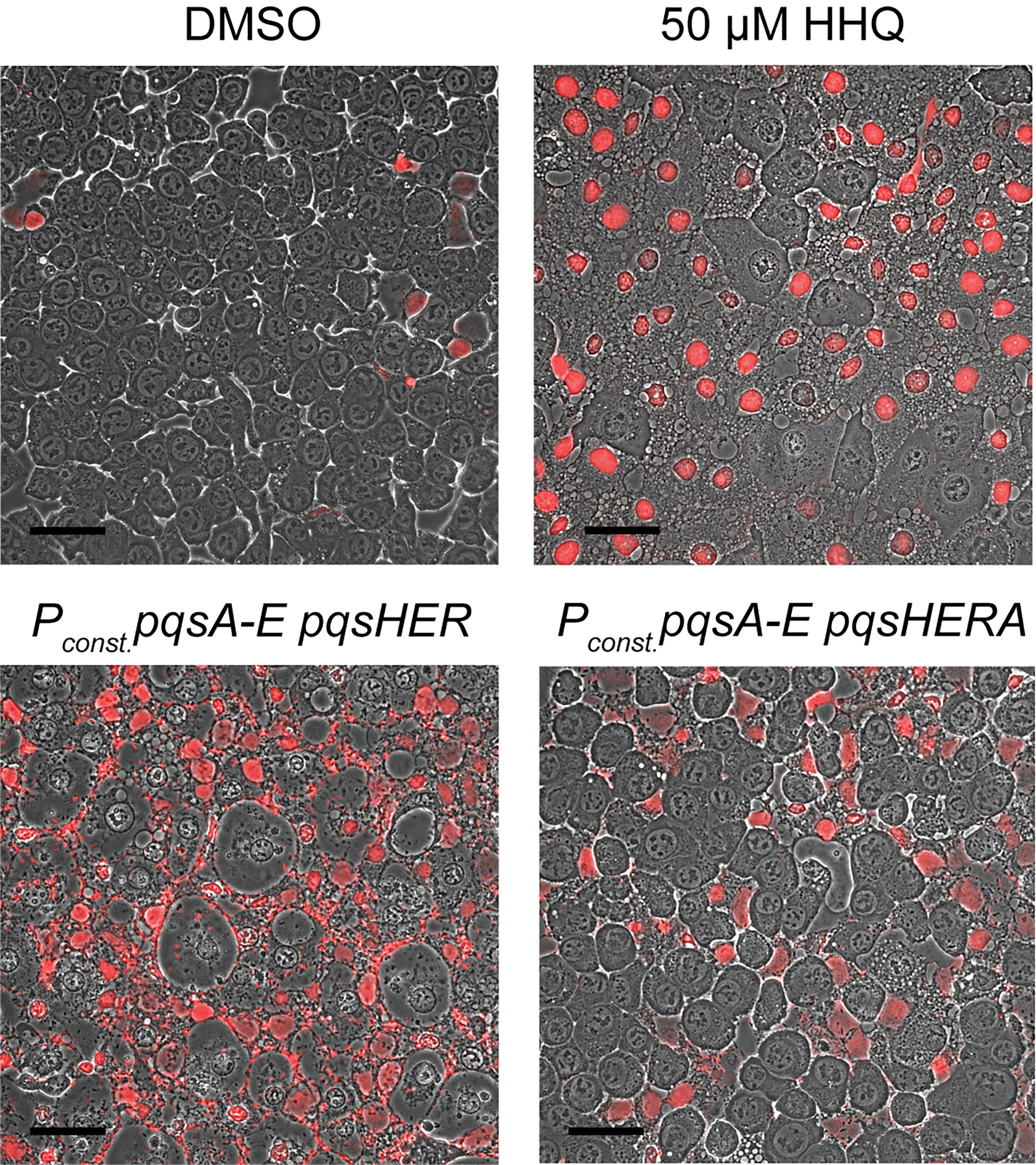

Supplement: S6 Fig — Representative images of TIB-67 monocytes co-cultured with surface-attached P. aeruginosa or treated with exogenous HHQ under conditions of the microscopy-based virulence assay (t = 16 h). Propidium iodide (PI) staining of DNA of non-viable cells indicates cell death (scale bars = 50 μm). (TIF) [file ppat.1008867.s008.tif]

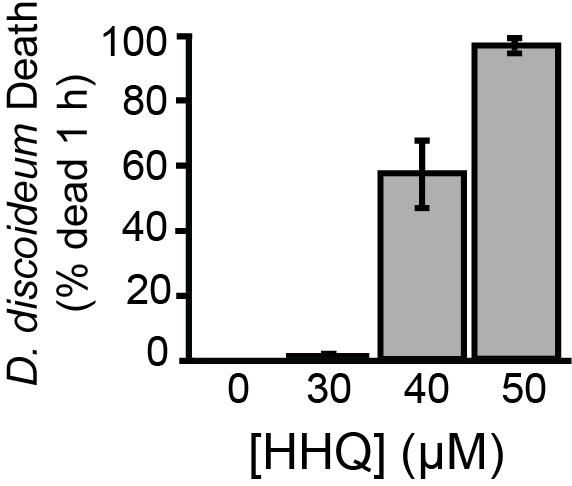

Supplement: S7 Fig — Cytotoxicity of purified HHQ towards D. discoideum under conditions of the P. aeruginosa virulence assay. HHQ was added to 1% agar pads used for imaging. Values are mean ± SEM of three biological replicates (n = 3). Approximately 200–300 cells were analyzed for each measurement. (TIF) [file ppat.1008867.s009.tif]

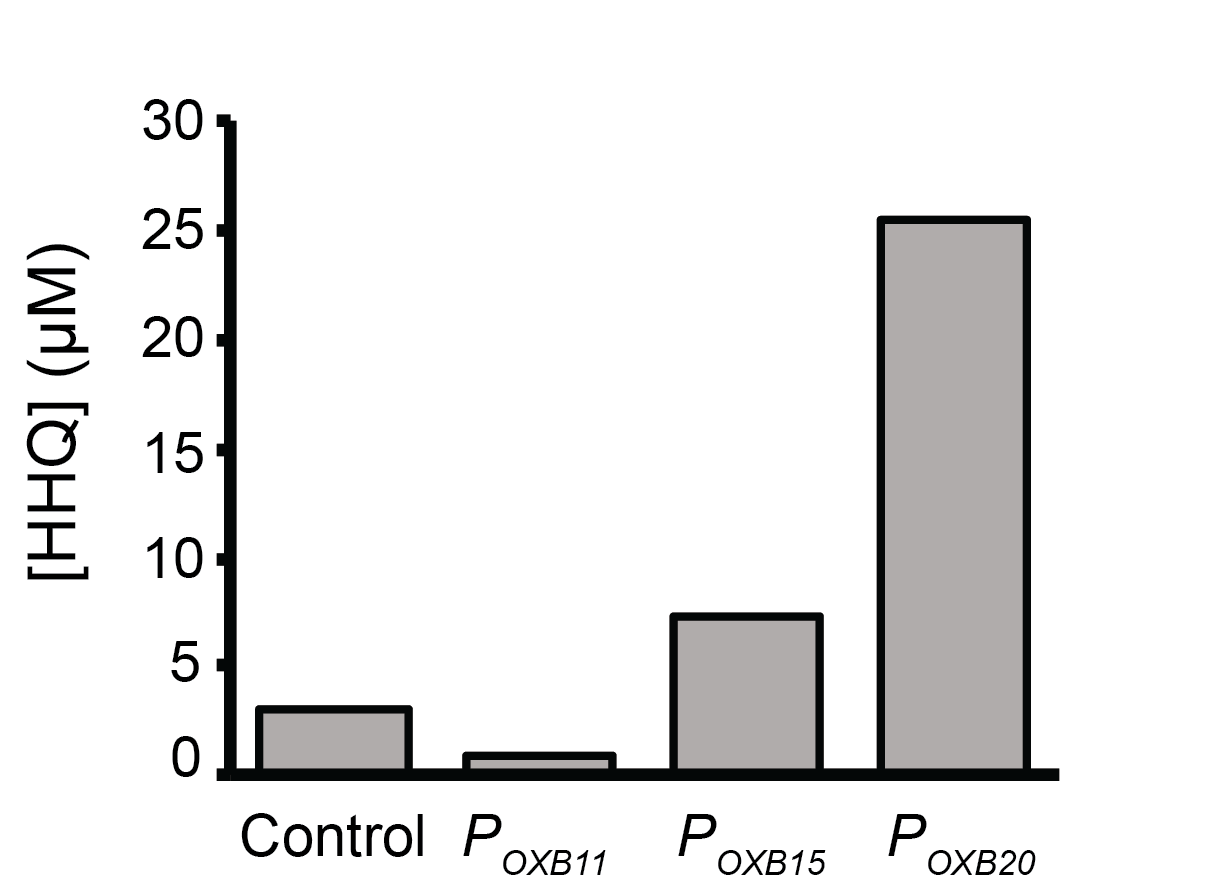

Supplement: S8 Fig — AQ biosensor-based quantification of HHQ levels in surface-attached pqsH P. aeruginosa populations. Expression of pqsABCDE genes are controlled by a constitutive promoter with high (POXB20), moderate (POXB15), or low (POXB11) levels of expression. Mean biosensor signal was calculated from >2,500 cells and converted to HHQ levels using a standard curve constructed using purified HHQ standards. (TIF) [file ppat.1008867.s010.tif]

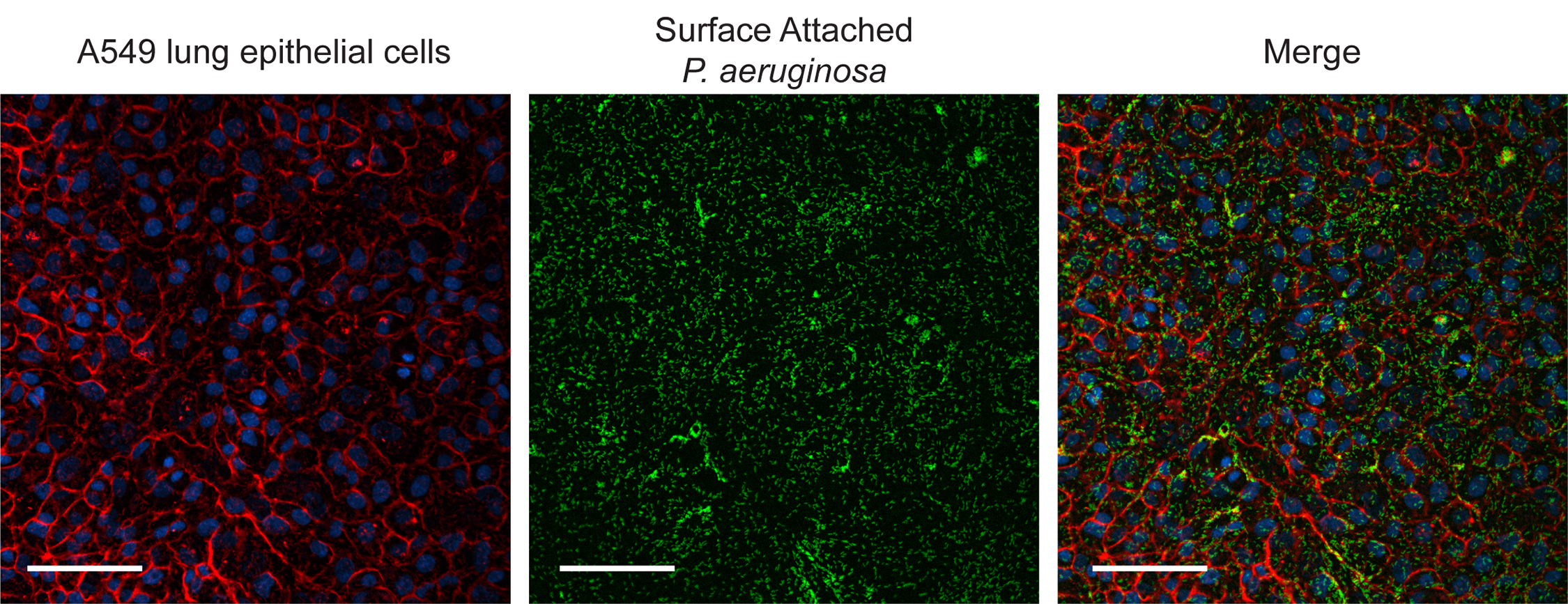

Supplement: S9 Fig — Confirmation of bacterial attachment to monolayers of A549 human lung epithelial cells. Wild type P. aeruginosa expressing constitutive oprM::mNeonGreen was grown to OD = 0.6 in DMEM and transferred to confluent monolayers of A549 cells. Co-cultures were grown for 1 h at 37°C with shaking (80 rpm), then monolayers were washed twice with DPBS, treated briefly with FM 4–64 and Hoescht, then covered with 1% agar pad prepared with PBS. Fluorescent images were taken immediately (scale bars = 100 μm) (TIF) [file ppat.1008867.s011.tif]

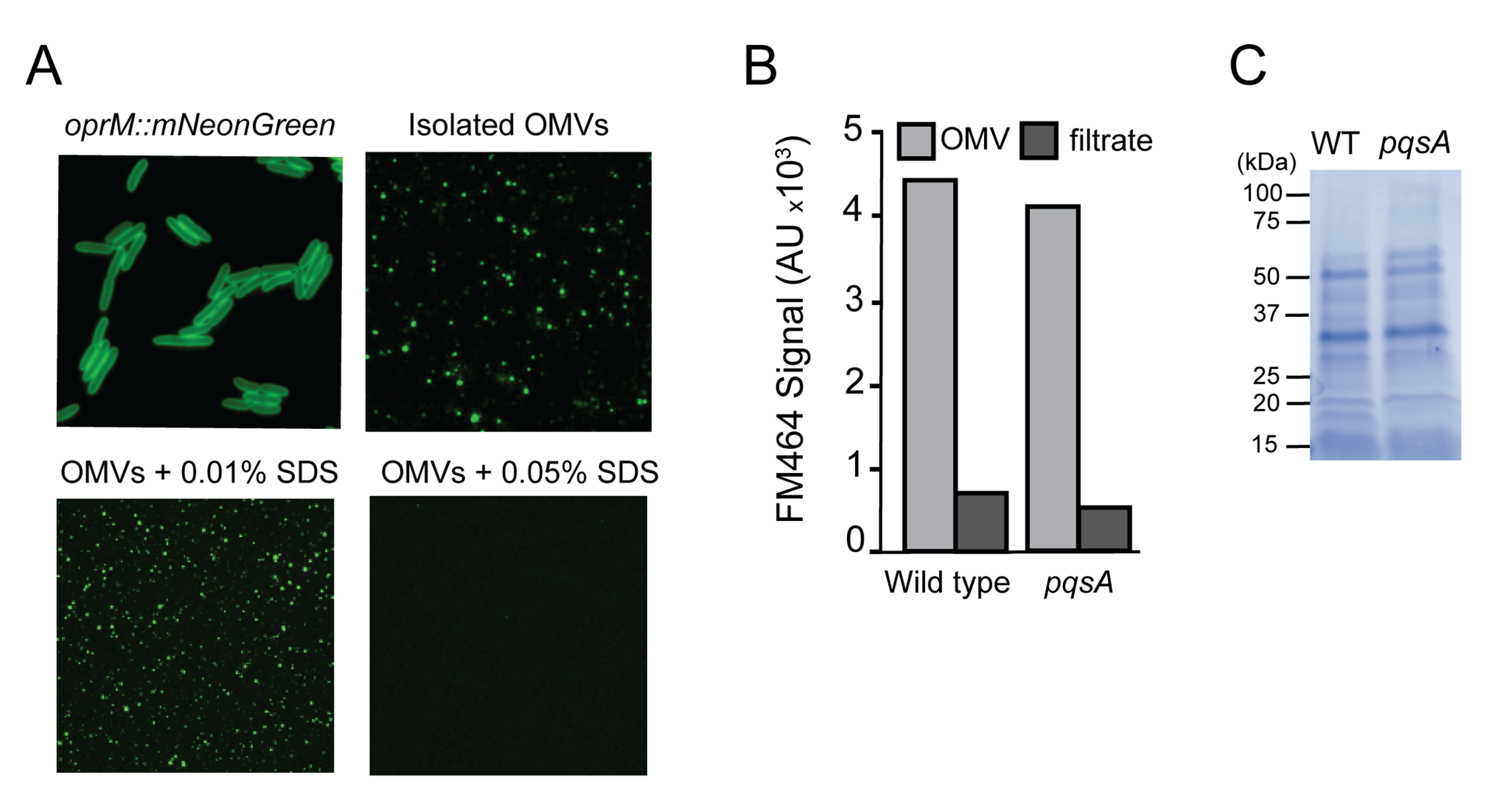

Supplement: S10 Fig — Isolation and lysis of fluorescently labelled outer membrane vesicles (OMVs) from P. aeruginosa. (A) Representative images of POXB20-oprM::mNeonGreen P. aeruginosa cells and OMVs isolated from supernatants of this strain following procedures described in Materials and Methods. OMVs were treated with 0.05% SDS at 37°C for 1 h to induce OMV lysis and subsequent diffusion of the fluorescent signal. SDS concentrations lower than 0.05% were not sufficient to cause lysis. Fluorescent signal does not accurately reflect OMV size (generally < 200 nm diameter) due to the diffraction limit of light microscopy (scale bars = 20 μm) (B) Representative measurements of OMV levels in samples isolated from WT and pqsA P. aeruginosa using FM464-based quantification method described in Materials and Methods. FM464 signal is fluorescence at (ex = 515 em = 645) in arbitrary fluorescence units. Filtrate is eluent obtained from concentrating OMV samples in 100 MWCO centrifugal concentration units. (C) Protein content of WT and pqsA OMVs analyzed by SDS-PAGE and Coomasie staining. (TIF) [file ppat.1008867.s012.tif]

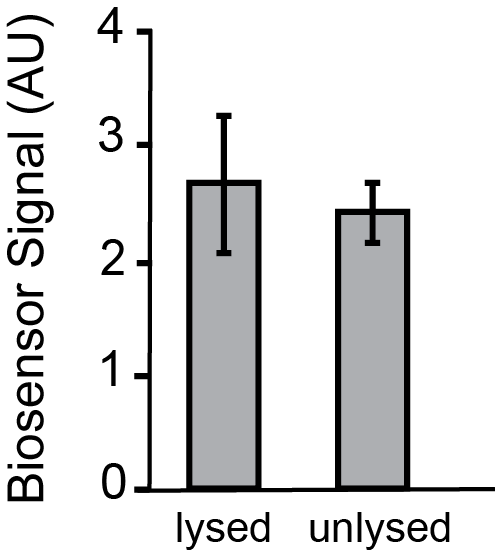

Supplement: S11 Fig — Treatment of AQ biosensor with lysed and unlysed OMV samples isolated from WT P. aeruginosa. OMV stocks isolated from non-fluorescent bacteria were added to biosensor cells in 96-well format, and YFP and mKate fluorescence were measured after 2 h incubation at 37°C 450 rpm. YFP/mKate fluorescence was subtracted by the mean biosensor signal of a sample treated with unlysed OMVs isolated from a pqsA mutant. Values shown are mean and error bars represent SD (n = 5). Dilution show is within the linear range of the AQ biosensor as shown in Fig 2A. (TIF) [file ppat.1008867.s013.tif]

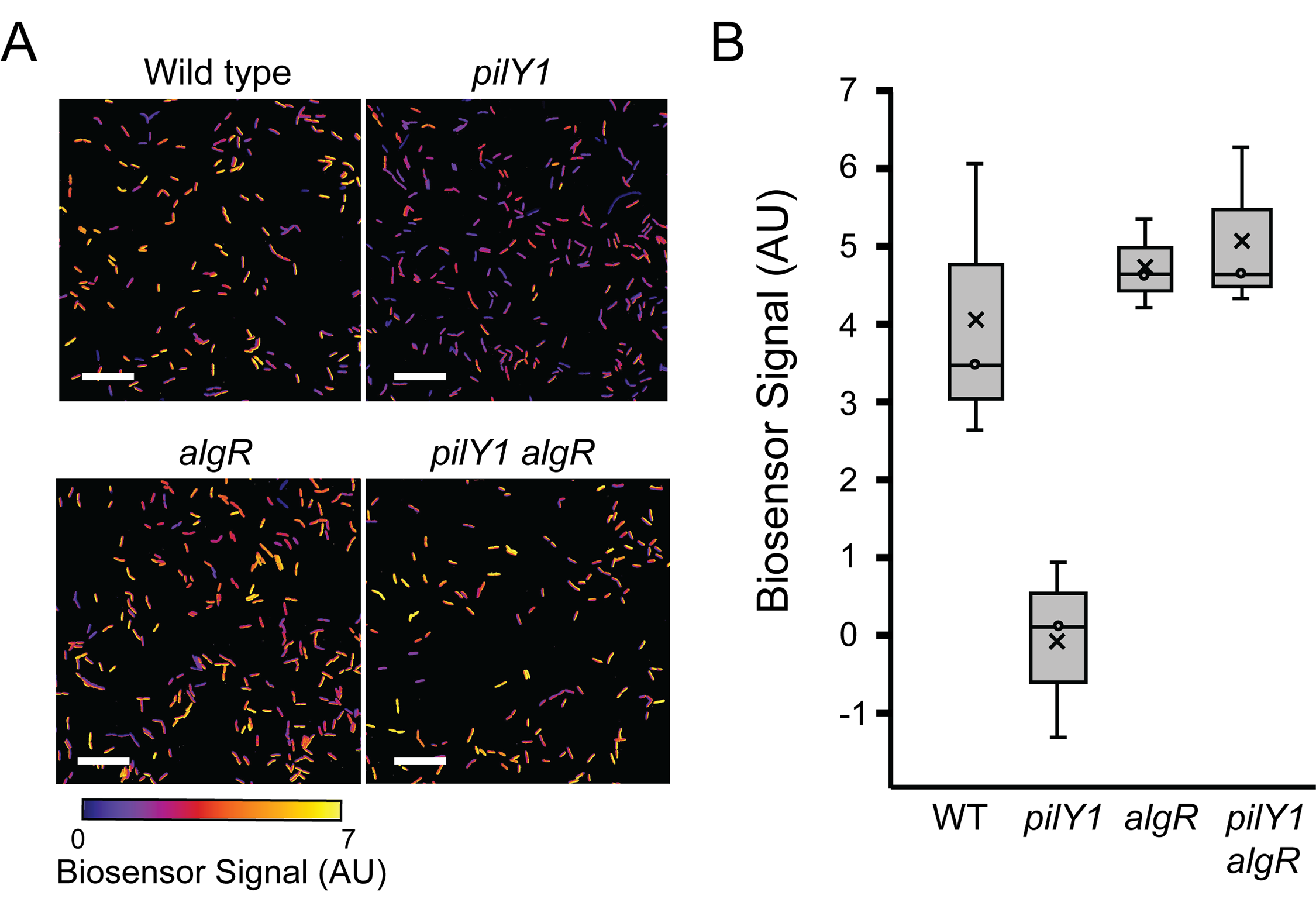

Supplement: S12 Fig — Biosensor-based quantification of AQ levels in surface-attached P. aeruginosa populations. (A) Representative images of AQ biosensor doped (1:100) into P. aeruginosa samples as described in Fig 2 (scale bars = 10 μm). (B) Quantification of biosensor signal described in (A). Mean YFP/mKate fluorescence intensity per cell was calculated for approximately 500 cells, and values were baseline subtracted by the mean YFP/mKate fluorescence of biosensor cells doped into the surface-attached pqsA mutant. Values are averages of three independent experiments and error bars represent standard error. (TIF) [file ppat.1008867.s014.tif]
