# Supplementary material for: Cytotoxic alkyl-quinolones mediate surface-induced virulence in Pseudomonas aeruginosa
Source: PLoS Pathog. 2020 Sep 14;16(9):e1008867. doi: 10.1371/journal.ppat.1008867 (PMC7515202; doi:10.1371/journal.ppat.1008867)
Supplement: S1 Table — (DOCX) [file ppat.1008867.s015.docx]

| **S1 Table. Bacterial strains and cell lines used in this study.** | | |  |
| --- | --- | --- | --- |
| **Parent Strain** | **Strain** | **Relevant Characteristics** | **Source** |
| *P. aeruginosa* PA14 | Wild type | - | [1] |
|  | *lasR* | *ΔlasR* | [2] |
|  | *pilY1* | *ΔpilY1* | [3] |
|  | *pqsA* | *ΔpqsA* | This study |
|  | *pqsE* | *ΔpqsE* | This study |
|  | *pqsH* | *ΔpqsH* | This study |
|  | *pqsR* | *ΔpqsR* | This study |
|  | *pqsL* | *pqsL::Mar2xT7* | [4] |
|  | *algR* | *ΔalgR* | This study |
|  | *algR pilY1* | *ΔalgR ΔpilY1* | This study |
|  | *algR pilY1 rhlR* | *ΔalgR ΔpilY1 rhlR::Mar2xT7* | This study |
|  | *phz* | Δ*phzABCDEFG1 ΔphzABCDEFG2* | This study |
|  | *rhlB* | *rhlB::Mar2xT7* | [4] |
|  | *hcnB* | *hcnB::Mar2xT7* | [4] |
|  | *rsmA* | *ΔrsmA* | This study |
|  | *gacA* | *ΔgacA* | This study |
|  | *vfr sadC* | *Δvfr ΔsadC* | [5] and this study |
|  | *pvdS fpvI* | *ΔpvdS ΔfpvI* | This study |
|  | *P_const_-pqsA-E* | *P_OXB20_::pqsA* | This study |
|  | *P_const_-pqsA-E pqsR* | *P_OXB20_::pqsA ΔpqsR* | This study |
|  | *P_const_-pqsA-E pqsH* | *P_OXB20_::pqsA ΔpqsH* | This study |
|  | *P_const_-pqsA-E pqsHER* | *P_OXB20_::pqsA ΔpqsH ΔpqsE ΔpqsR* | This study |
|  | *P_const_-pqsA-E pqsHERA* | *P_OXB20_::pqsA ΔpqsH ΔpqsE ΔpqsR pqsA::Mar2xT7* | This study |
|  | *P_const_-pqsA-E lasR* | *P_OXB20_::pqsA ΔlasR* | This study |
|  | *POXB15-pqsA-E* | *P_OXB15_::pqsA ΔpqsH* | This study |
|  | *POXB11-pqsA-E* | *P_OXB11_::pqsA ΔpqsH* | This study |
|  | *P_pqsA_-mCherry* | *attB::P_pqsA_-mCherry* | This study |
|  | *P_pqsA_-mCherry pilY1* | *attB::P_pqsA_-mCherry ΔpilY1* | This study |
|  | *P_pqsA_-mCherry lasR* | *attB::P_pqsA_-mCherry ΔlasR* | This study |
|  | *Plrs1-mCherry* | *attB::Plrs1-mCherry* | This study |
|  | *Plrs1-mCherry rhlR* | *attB::Plrs1-mCherry rhlR::Mar2xT7* | This study |
|  | *P_lac_-vector* | pBBRMCS3 | [6] |
|  | *P_lac_-algR^D54E^* | pBBRMCS3::*P_lac_-algR(D54E)* | This study |
| *P. aeruginosa* PAO1 | PAO1 Biosensor | *ΔpqsA ΔpqsR glmS::P_tac_-pqsR* pUCP18*::P_rpoD_-mKate P_pqsA_-YFP* | This study |
| *E. coli* B/r | Wild type | - | [7] |
| *D. discoidium* | AX3 | *-* | [8] |
| *M. musculus* | J774A.1(ATCC® TIB-67™) | *-* | ATCC |

**References**

1. Rahme LG, Stevens EJ, Wolfort SF, Shao J, Tompkins RG, Ausubel FM. Common virulence factors for bacterial pathogenicity in plants and animals. Science. 1995;268(5219):1899-902.
2. O’Loughlin CT, Miller LC, Siryaporn A, Drescher K, Semmelhack MF, Bassler BL. A quorum-sensing inhibitor blocks *Pseudomonas aeruginosa* virulence and biofilm formation. Proc Natl Acad Sci USA. 2013;110(44):17981-6.
3. Kuchma SL, Delalez NJ, Filkins LM, Snavely EA, Armitage JP, O'Toole GA. Cyclic di-GMP-mediated repression of swarming motility by *Pseudomonas aeruginosa* PA14 requires the MotAB stator. J Bacteriol. 2015;197(3):420-30.
4. Liberati NT, Urbach JM, Miyata S, Lee DG, Drenkard E, Wu G, et al. An ordered, nonredundant library of *Pseudomonas aeruginosa* strain PA14 transposon insertion mutants. Proc Natl Acad Sci USA. 2006;103(8):2833-8.
5. Siryaporn A, Kuchma SL, O’Toole GA, Gitai Z. Surface attachment induces *Pseudomonas aeruginosa* virulence. Proc Natl Acad Sci USA. 2014;111(47):16860-5.
6. Kovach ME, Elzer PH, Hill DS, Robertson GT, Farris MA, Roop II RM, et al. Four new derivatives of the broad-host-range cloning vector pBBR1MCS, carrying different antibiotic-resistance cassettes. Gene. 1995;166(1):175-6.
7. Fey P, Kowal AS, Gaudet P, Pilcher KE, Chisholm RL. Protocols for growth and development of *Dictyostelium discoideum*. Nat Protoc. 2007;2(6):1307
8. Loomis Jr WF. Sensitivity of *Dictyostelium discoideum* to nucleic acid analogues. Exp Cell Res. 1971;64(2):484-6.
