# Supplementary material for: Cytotoxic alkyl-quinolones mediate surface-induced virulence in Pseudomonas aeruginosa
Source: PLoS Pathog. 2020 Sep 14;16(9):e1008867. doi: 10.1371/journal.ppat.1008867 (PMC7515202; doi:10.1371/journal.ppat.1008867)
Supplement: S2 Table — (DOCX) [file ppat.1008867.s016.docx]

| **S2 Table. Plasmids used in this study.** | |  |
| --- | --- | --- |
| **Parent Strain** | **Plasmid** | **Source** |
| *E. coli* S17 | pEXG2 | [1] |
|  | mini-CTX-2 | [2] |
|  | pUC18-mini-Tn7T-Gm | [3] |
|  | pUC18-mini-Tn7T-LAC | [3] |
|  | pUCP18-RedS | [4] |
|  | pFLP2 | [3] |
|  | pUCP18::*P_rpoD_-mKate P_PaQa_-YFP* | [5] |
|  | mini-CTX-2::*P_tac_-mCherry* | [6] |
|  | pBBRMCS3 | [7] |
|  | pSF-OXB20 | Oxford Genetics |
|  | pSF-OXB15 | Oxford Genetics |
|  | pSF-OXB11 | Oxford Genetics |
|  | pUC19 | New England Biolabs |

**References**

1. Hmelo LR, Borlee BR, Almblad H, Love ME, Randall TE, Tseng BS, et al. Precision-engineering the *Pseudomonas aeruginosa* genome with two-step allelic exchange. Nat Protoc. 2015;10(11):1820.
2. Hoang TT, Kutchma AJ, Becher A, Schweizer HP. Integration-proficient plasmids for *Pseudomonas aeruginosa*: site-specific integration and use for engineering of reporter and expression strains. Plasmid. 2000; 43(1):59– 72
3. Hoang TT, Karkhoff-Schweizer RR, Kutchma AJ, Schweizer HP. A broad-host-range Flp-FRT recombination system for site-specific excision of chromosomally-located DNA sequences: application for isolation of unmarked *Pseudomonas aeruginosa* mutants. Gene. 1998;212(1):77-86.
4. Lesic B, Rahme LG. Use of the lambda Red recombinase system to rapidly generate mutants in *Pseudomonas aeruginosa*. BMC Mol Biol. 2008;9(1):20.
5. Persat A, Inclan YF, Engel JN, Stone HA, Gitai Z. Type IV pili mechanochemically regulate virulence factors in *Pseudomonas aeruginosa*. Proc Natl Acad Sci USA. 2015:201502025.
6. Siryaporn A, Kim MK, Shen Y, Stone HA, Gitai Z. Colonization, competition, and dispersal of pathogens in fluid flow networks. Curr Biol. 2015;25(9):1201-7.
7. Rao X, Huang X, Zhou Z, Lin X. An improvement of the 2ˆddCT method for quantitative real-time polymerase chain reaction data analysis. Biostat Bioform Biomath. 2013;3(3):71.
