# Supplementary material for: Cytotoxic alkyl-quinolones mediate surface-induced virulence in Pseudomonas aeruginosa
Source: PLoS Pathog. 2020 Sep 14;16(9):e1008867. doi: 10.1371/journal.ppat.1008867 (PMC7515202; doi:10.1371/journal.ppat.1008867)
Supplement: S3 Table — (DOCX) [file ppat.1008867.s017.docx]

| **Table S3. Primers used in this study.** | |  |
| --- | --- | --- |
| Primer | Sequence |  |
| pqsUP-5 | GAT ACA AAG CTT TCC AAC CGC CCG TAC TGC | |
| pqsUP-3 | GCA CAC GGC GTT TCT ACA TAG CTG CCA TTT GCA GGC CTC C | |
| pqsDOWN-5 | GGA GGC CTG CAA ATG GCA GCT ATG TAG AAA CGC CGT GTG C | |
| pqsDOWN-3 | CGT TCC CTC TTC AGC GAT ATG GGG TGT GTC GAG TGG ATG G | |
| OXB20-5 | CCA TCC ACT CGA CAC ACC CCA TAT CGC TGA AGA GGG AAC G | |
| OXB20-3 | GAT ACA AAG CTT CCA GCG AGA AAT CGT CGA GC | |
| pqsA-PaQa-5 | GAT ACC CTC GAG GTG GGT GTG CCA AAT TTC TCG | |
| pqsA-PaQa-3 | GAT ACA GGA TCC CAG CGA TAT GCA TCC GGA TCA G | |
| pqsR-5 | GAT ACC GCT AGC GAC CCG ATC AAG GGA AGC G | |
| pqsR-3 | GAT ACC GCT AGC GCT CTA CTC TGG TGC GGC | |
| pqsR-KO1 | GAT ACA AAG CTT CCT CAC CTC CAA AAC GAC G | |
| pqsR-KO2 | GCG CCT TCG GGC CTG AGG CGG AGG AAA TCG AAC CGG | |
| pqsR-KO3 | CCG GTT CGA TTT CCT CCG CCT CAG GCC CGA AGG CGC | |
| pqsR-KO4 | GAT ACA AAG CTT GCT GGA ATT GCT CGC CTG G | |
| algR-KO1 | GAT ACA AAG CTT ACC TGT CCG ACC TGT TCC G | |
| algR-KO2 | GCA TCA GAC GCC TGA CCC CGC CAG AGG TTC GTC ATC GAC | |
| algR-KO3 | GTC GAT GAC GAA CCT CTG GCG GGG TCA GGC GTC TGA TGC | |
| algR-KO4 | GAT ACA AAG CTT GCT CGA GGC TGG CGT AGG | |
| pqsA-KO1 | GAT ACA AAG CTT GCC TCG AAC TGT GAG ATC TGG | |
| pqsA-KO2 | CGT GAT AAA GGG TGT CGG CCG GTC AGG TTG GCC AAT GTG G | |
| pqsA-KO3 | CCA CAT TGG CCA ACC TGA CCG GCC GAC ACC CTT TAT CAC G | |
| pqsA-KO4 | GAT ACA AAG CTT GAC CAG GAC GTT GCG ATA GC | |
| pqsE-KO1 | GAT ACA AAG CTT CCT TCC TCG ATG AGA ACG TCC | |
| pqsE-KO2 | GCT CCC CAG GTG CAG TTC GTC ATC ATC CAG TTG ACC GGG | |
| pqsE-KO3 | CCC GGT CAA CTG GAT GAT GAC GAA CTG CAC CTG GGG AGC | |
| pqsE-KO4 | GAT ACA AAG CTT CTC AAC GGT GCC AGC AAG G | |
| pqsH-KO1 | GAT ACA AAG CTT AGC GGG GTC TGC GTA TAG C | |
| pqsH-KO2 | TAC TGT GCG GCC ATC TCA CCC TGG ATA AGA ACG GTC ATC CG | |
| pqsH-KO3 | CGG ATG ACC GTT CTT ATC CAG GGT GAG ATG GCC GCA CAG TA | |
| pqsH-KO4 | GAT ACA AAG CTT CAG TCT TCA CCG CAG TCG G | |
| algR-pUC19-5 | GAT ACA GGT ACC ATG AAT GTC CTG ATT GTC GAT GAC | |
| algR-pUC19-3 | GAT ACC GAG CTC TCA GAG CTG ATG CAT CAG ACG | |
| algR-D54E-Fw | CCC GAT ATC GTC CTG CTG GAA ATC CGC ATG CCC GGT CTG G | |
| algR-D54E-Rv | CCA GAC CGG GCA TGC GGA TTT CCA GCA GGA CGA TAT CGG G | |
| algR-pBBR-5 | GAT ACA GGT ACC TCA TGC AGG AAG CCT GAG CT | |
| algR-pBBR-5 | GAT ACC GAG CTC TCA GAG CTG ATG CAT CAG ACG | |
| PpqsA-1 | GAT ACA AAG CTT AGG CCT GCA AAT GGC AGG | |
| PpqsA-2 | CTC GCC CTT GCT CAC CAT GAC AGA ACG TTC CCT CTT CAG C | |
| PpqsA-3 | GCT GAA GAG GGA ACG TTC TGT CAT GGT GAG CAA GGG CGA G | |
| PpqsA-4 | CCC GGG CTG CAG GAA TTC | |
| Plrs1-1 | GAT ACA AAG CTT GCT GCC AAA GAA TCG CGA CC | |
| Plrs1-1 | CAC CAT GCT TAA TTT CTC CTC TTT AAA CCA ATG ACA ACC CAC TTT GC | |
| Plrs1-3 | GCA AAG TGG GTT GTC ATT GGT TTA AAG AGG AGA AAT TAA GCA TGG TG | |
| Plrs1-4 | GAT ACA AAG CTT CTA CTT GTA CAG CTC GTC CAT G | |
| oprM-Neon-1 | GAT ACA AAG CTT CGT CCG TCG AAA AGG GGC | |
| oprM-Neon-2 | GTG AGC AAG GGC GAG GAG GAT AAC GGT GTT CTG CCC GTA GGC | |
| oprM-Neon-3 | GCC TAC GGG CAG AAC ACC GTG AGC AAG GGC GAG GAG GAT AAC | |
| oprM-Neon-4 | GAT ACA GCT AGC TTA CTT GTA CAG CTC GTC CAT GCC | |
| oprM-Neon-5 | GCT GGC CTT TTG CTC ACA TGG CTC GAT CGC TTG ATA AGG TCC | |
| oprM-Neon-6 | ACG ACG GCC AGT GCC AAG CTG TAT CAG TCA GTCA GTG CAG GAG G | |
| pqsA-lrs1-Fw | CGA ATA TCG CGC TTC GCC C | |
| pqsA-lrs1-Rv | GGT CAG GTT GGC CAA TGT GG | |
| pqsA-Fw | GTG CCT TCC ATC GCC AGG | |
| pqsA-Rv | CAG CAA CTC CGT AGC GAA CG | |
| gapA-Fw | CTG CCC TGG AAG AGC CTC G | |
| gapA-Rv | CAG CAC CTC GTG GTT GAC G | |
