# Supplementary material for: Cytotoxic alkyl-quinolones mediate surface-induced virulence in Pseudomonas aeruginosa
Source: PLoS Pathog. 2020 Sep 14;16(9):e1008867. doi: 10.1371/journal.ppat.1008867 (PMC7515202; doi:10.1371/journal.ppat.1008867)
Supplement: Attachment — Submitted filename: Response to Reviewers.docx [file ppat.1008867.s019.docx]

 Dear Dr. Gitai,
   
    Thank you very much for submitting your manuscript "Cytotoxic alkyl-quinolones mediate surface-induced virulence in Pseudomonas aeruginosa" (PPATHOGENS-D-19-02344) for consideration at PLOS Pathogens. As with all papers peer reviewed by the journal, your manuscript was reviewed by members of the editorial board and by several independent peer reviewers. Based on the reports, we regret to inform you that we will not be pursuing this manuscript for publication at PLOS Pathogens.
   
    Three experts in Pseudomonas biology were retained to review the manuscript. All three agreed that the manuscript was nicely written and the experiments were well designed. They were particularly impressed with the genetic approaches. Unfortunately, two of the three reviewers had major concerns; 1) regarding the novelty of the findings given the existing literature and 2) the biological significance of the data and conclusions given the high concentrations HHQ needed to recapitulate the killing phenotype.
   
   
    The reviews are attached below this email, and we hope you will find them helpful if you decide to revise the manuscript for submission elsewhere. We are sorry that we cannot be more positive on this occasion. We very much appreciate your wish to present your work in one of PLOS's Open Access publications.
   
    Thank you for your support, and we hope that you will consider PLOS Pathogens for other submissions in the future.
   
   
    Sincerely,
   
    Alan Hauser
    Section Editor
    PLOS Pathogens
   
    Kasturi Haldar
    Editor-in-Chief
    PLOS Pathogens
    orcid.org/0000-0001-5065-158X
   
    Michael Malim
    Editor-in-Chief
    PLOS Pathogens
    orcid.org/0000-0002-7699-2064
   
   
    **************************************
    Reviewer's Responses to Questions
   
    <b>Part I - Summary</b></br>Please use this section to discuss strengths/weaknesses of study, novelty/significance, general execution and scholarship.<br/><br/>
   
    Reviewer #1: In this study, Vrla et al. used a combination of genetics, cell biology, and imaging to demonstrate that surface-induced virulence of Pseudomonas aeruginosa, a phenomenon the group first described in 2014 (Siryaporn et al. 2014), is mediated by alkyl-quinolones. They show that the AQs HHQ and PQS elicit cytotoxicity in Dictyostelium, and that the previously identified contact-dependent regulators LasR and PilY induce AQs via activation of pqsA. They further show that the AQ HHQ is also cytotoxic towards mammalian macrophages.
    Overall, this is a well-written manuscript that further elucidates the specific virulence mechanism induced by P. aeruginosa surface association to target a broad range of host cells, and explains the joint regulation of surface-induced virulence by both LasR and PilY. The experimental work is extensive and rigorous, but there are some sections that would benefit from re-organization, and some points that need clarification, as detailed below.

**We thank the reviewer for their kind words about the paper and its significance. We have restructured the text as suggested to aid clarity and appreciate the constructive suggestions.**
   
    Reviewer #2: Previous studies revealed that P. aeruginosa cytotoxicity becomes activated upon bacterial attachment to solid surfaces. It was demonstrated that cytotoxicity requires functional pilY1 and lasR genes. However, the nature of the cytotoxin has remained unknown.
    In this study the authors demonstrate that the P. aeruginosa alkylquinolones (AQ) have a cytotoxic activity towards eukaryotic cells (D. discoideum and to a lower extend - mammalian monocytes) and that the expression of the pqsA-E operon - involved in AQ biosynthesis - is induced in a pilY1 and lasR dependent manner upon surface attachment.
    It is well established that the AQs exert cytotoxic effects on host cells and that the pqsA-E AQ biosynthetic operon is induced by LasR. What is new is that surface attachment activates directly or indirectly LasR and/or PilY1, to induce pqsA-E expression. Unfortunately, the present study is restricted to the finding that pqsA-E transcription is induced upon attachment and that AlgR acts downstream of PilY1. No attempts to uncover the role of surface attachment in the activation of the LasR and/or PliY1 system, or how AlgR regulates pqsA-E expression are made.
    The manuscript is well written, easy to follow, and the experiments are sound. However, I do find the current manuscript lacking in sufficient novelty of results.

**We share the reviewer’s interest in determining the mechanism by which surface activation induces AQs, but this is a separate project that is beyond the scope of the current work (which we and both Reviewers 1 and 3 find significant on its own).**

**Nevertheless, recognizing the reviewer’s point, we have attempted to bring an additional new finding to light in adding a new section on how outer membrane vesicles (OMVs) enhance the cytotoxic activity of AQs. These new data both strengthen the physiological significance of our findings (addressing Reviewer 3’s concern below) and add to the novel findings in the current paper.**

**Finally, we have answered the reviewer’s legitimate question concerning how AlgR regulates pqsA-E expression. Specifically, we now show that AlgR regulates RhlR, which was previously thought to repress pqs expression, but can actually induce pqs expression in surface-activated virulence. We explore the mechanistic basis of this and show that the sRNA Lrs1 upstream of the pqsA gene, which our lab recently showed is surface induced, is positively regulated by RhlR. This upstream promoter expression can repress the pqs expression if terminated (explaining the previously published results), but here we show that it can also lead to run-on transcription that induces pqs operon expression.**
   
    Reviewer #3: The manuscript by Vrla et. al. investigates the role of alkyl-quinolones produced by P. aeruginosa in the increased virulence observed when the organism is attached to a surface. The authors provide strong genetic evidence identifying a role for AQs in this phenotype, but I have concerns about the physiological relevance of the biochemical experiments (exogenous addition of AQs) and therefore the prominent claim of AQ sufficiency to explain the observed phenotype.

**We thank the reviewer for pointing out the important discrepancy between the in vivo and in vitro levels of AQs. We have now provided a physiological explanation for these differences: in vivo, AQs are packaged into OMVs, which significantly potentiate their activity, explaining why they are active at such lower levels. Furthermore, while OMVs have long been known to be cytotoxic, the toxins mediating that cytotoxicity were not identified and we now show that OMVs lacking AQs lose cytotoxicity. These new findings thus simultaneously provide significant new insights into AQs as mediators of OMV cytotoxicity and bolster the physiological significance of AQs as cytotoxins.**
   
    --------------------
   
    <b>Part II – Major Issues: Key Experiments Required for Acceptance</b></br>Please use this section to detail the key new experiments or modifications of existing experiments that should be <u>absolutely</u> required to validate study conclusions.<br/><br/>Generally, there should be no more than 3 such required experiments or major modifications for a "Major Revision" recommendation. If more than 3 experiments are necessary to validate the study conclusions, then you are encouraged to recommend "Reject".<br/><br/>
   
    Reviewer #1: N/A
   
    Reviewer #2: (No Response)
   
    Reviewer #3: - A major concern is the very high concentration of HHQ required to see any effect on target cells.  Fig 1D shows that 50 uM HHQ is required to match the killing phenotype seen with bacteria in panel C, and no effect at all is seen until 40 uM HHQ is used.  The authors cited Xiao 2006 (who measured 20 uM HHQ) to claim that 50 uM HHQ is physiologically relevant.  That is the highest reported concentration of HHQ that I could find.  A survey of other reports (Deziel 2004, Lesic 2007, Lepine 2007, Hazan 2010, Lamarche 2011, Fito-Boncompte 2011 and Hazan 2016) provide an average physiological concentration of HHQ of ~7.5 uM.  The authors’ own data (Fig S2, Fig 2D) shows HHQ production of approx. 2 and 3.5 uM, respectively.  Even the pqsH mutant (known in the literature to dramatically overproduce HHQ) produced only 20 uM HHQ as measured by indirect biosensor assay (Fig 3E).  I am concerned that the exogenous HHQ addition experiments are not physiologically relevant
    and therefore that the claim that direct killing by AQs (and specifically HHQ) is sufficient to explain the observed phenotype with live cells in invalid.

**We thank the reviewer for bringing up this important point. First of all, we want to emphasize that while 50 uM PQS is necessary to kill *D. discoideum* rapidly (within 1 hour), we also show that much lower levels (1 uM) are sufficient to inhibit *D. discoideum* growth and to kill mammalian monocytes, demonstrating that the purified compounds do have efficacy alone at lower levels. More importantly, we now directly address the reviewer’s concern by demonstrating that AQs are more potent when produced by *P. aeruginosa* than in pure form due to their packaging in OMVs (Fig. 3), showing that the levels of AQ reported as physiological in the literature are sufficient to induce killing when produced by bacteria. Together these findings support the physiological relevance of our original claim that AQs act as surface-induced cytotoxins and we thank the reviewer for pushing us to clarify this important issue.**

    - The claim of sufficiency is also challenged by the results of the more disease-relevant experiments performed with mammalian cells.  In this system (Fig 4B), the pqsA mutant bacteria still killed ~60% of the mammalian cells.  The authors comment that there must be other factors that contribute to the killing of mammalian cells, which means that AQ cytotoxicity is not sufficient to explain this killing.

**We attempt to be very clear about the fact that our findings demonstrate that AQs alone can kill both *D. discoideum* and monocytes (Fig. 1) but that these two systems differ in that loss of AQ production eliminates virulence towards *D. discoideum* but not towards monocytes. Thus, our claim is that AQs are sufficient to kill both cells but only necessary for *D. discoideum* killing. We discuss the fact that this suggests that toxicity towards monocytes is due to multiple redundant factors and discuss relevant candidates like T3SS.**
   
    - In the discussion, the authors bring up the possibility that relevant infections sites can be microaerophilic or even anoxic (which is true). This might lead one to look to the pqsH mutant scenario for parallels and then possibly reconsider the relevancy to the very high HHQ concentrations used in the experiments.  However, growth without oxygen is much different than growth without PqsH (in terms of global regulation among any number of other things), and HHQ production under anoxic conditions has previously been measured to be only 1.5 uM (Schertzer 2010).  This should be addressed if AQ production under microaerophilic/anoxic conditions is going to be discussed in support of physiological relevance.

**We thank the reviewer for this good point and have added it to the discussion.**
   
    -  Relating to the previous points, and to the general idea of AQs as secreted toxins discussed in this manuscript, it has been reported that total culture AQ extracts frequently to not well represent the amount of AQs actually secreted (Florez 2017, Alcalde-Rico 2018).  My reading of the methods in the current manuscript lead me to believe that the authors have reported total HHQ/PQS production in their experiments rather than secreted HHQ/PQS concentrations (which could be easily fixed by extracting the AQs from supernatants rather than whole culture).

**The reviewer is correct that the paper reports total AQ levels and not just secreted levels. Since both *D. discoideum* and monocytes are phagocytic and ingest the entire bacterium, we believe that this is the relevant concentration to consider. Nevertheless, acknowledging the reviewer’s point, we have clarified that we are only examining total AQ levels. In normal circumstances we would happily measure supernatant AQ levels but since the lab is currently closed due to the Covid-19 quarantine we do not know when we would be able to do these experiments. Since the results of the supernatant measurements would not significantly change our conclusions about AQ levels being surface-induced and important for surface-induced virulence, we believe that they should be considered beyond the scope of the claims of the current paper.**
   
    - I’m not sure how informative the constitutively-expressed pqsABCDE constructs are.  These constructs may be driving HHQ production to such high levels that they might, for instance, overcome the effect of the pqsH mutation seen in Fig 1C (left side) for this reason alone.  The concentrations of the AQs produced by these strains should be reported so that proper conclusions can be drawn as to the importance of PQS genes other than pqsA.

**To address this point we have generated two new strains that constitutively express *pqsABCDE* to lower levels (with weaker promoters) and measured their virulence and AQ levels using in a *pqsH* background. AQ levels increased with increasing expression (1 uM with the weakest promoter, 6 uM with the intermediate promoter, and 20 uM with the strongest promoter, Fig. S6). In a *pqsH* background, the weakest promoter produced virulence levels comparable to the *pqsH* mutant, and both the intermediate and strong promoters produced WT levels of virulence (Fig. S2). These results demonstrate that the virulence defect of the *pqsH* mutation does not required exorbitantly high levels of AQs.**
   
    - There are some unusual measurements for HHQ and PQS concentrations in some experiments.  Fig 2D shows twice as much HHQ produced as PQS.  This result contradicts a large body of literature and, in fact, the authors’ own data in Fig S2.  Fig 3E shows zero HHQ produced by planktonic delta-pqsH cells, which is also unusual since loss of PqsH usually greatly increases HHQ production.  These deviations from what is commonly seen in the literature should be mentioned in the discussion and any differences in experimental conditions or caveats in measurement techniques should be described.  I acknowledge that the authors do bring up Xiao 2006 where greater HHQ than PQS was measured in mouse burn wounds, but the experiments in the current manuscript were in vitro not in mice.

**We thank the reviewer for highlighting an important source of potential confusion. We observe that both the absolute and relative levels of AQs change in different conditions. In the original version of the manuscript we reported liquid-culture levels in three distinct states: early in stationary phase (OD ~ 1), in the liquid phase of the *D. discoideum* virulence assay, and late in stationary phase (OD ~ 4). The result the reviewer cites in Fig. 2D with more HHQ than PQS is due to the fact that these were measured in early stationary phase, and HHQ levels increase before PQS levels (PQS levels are strongly quorum-sensitive). Similarly, the levels of HHQ in the old Fig. 3E are lower than previously-reported values because we are measuring them at lower ODs. Since all of our virulence assays are performed at lower ODs, we believe that this is the relevant comparison. As a result, to make things simpler, we have eliminated the high-OD measurements from the revised version. We have also explained in the revised text how our measurements at lower ODs can explain the differences between our findings and previous reports (which we now better cite).**

    --------------------
   
    <b>Part III – Minor Issues: Editorial and Data Presentation Modifications</b></br>Please use this section for editorial suggestions as well as relatively minor modifications of existing data that would enhance clarity.<br/><br/>
   
    Reviewer #1: 1. Representative images of pgsA:cherry expression relative to the constitutive reporter in wild type, lasR, and pilY mutants should be included in the SI, to substantiate the quantitative data presented in Fig. 2C.

**Thank you – we have done this.**
   
    2. Figure 3 and S3 should be re-organized to enhance clarity: For Fig. 3A, it should be mentioned in the text and indicated in the graphs what the linear range is in each case, since the response is not linear over the whole range of concentrations shown in the graphs for either PQS or HHQ.

**Thank you – we have repeated these experiments several times and produced more reproducible standard curves, which we now show in new Figure 2D. We have also changed how we determine and explain the dynamic and linear ranges of our reporter. Our measurements show that in surface-attached cells, the levels of AQs we measure fall within the linear range of the standard curve. The levels of AQs in planktonic cells are below the detectable limit, so we report the detectable limit as their maximal value. This produces a lower bound on the fold induction of AQs by surface attachment at roughly 20-fold.**
   
    3. In Fig. 3B, a dose response for PQS is shown. This may be moved to the SI, since Fig. 3B is not mentioned in the Results section as far as I could see, and is not as relevant for the rest of the Figure as some of the data in Fig. S3, which may be moved to the main Fig. 3 instead.  The data for HHQ would be more relevant to show in the main figure 3, since HHQ is further quantified in 3D and 3E.

**Thank you – we have replaced these data with images of the response to HHQ, and included the original images of the PQS standard curve in Fig S7.**
   
    4. Fig. 3 legend: stats for 3D need to be corrected to (*) as indicated in the Figure panel. Stats for 3E need to be included.

**Thank you – we have done this.**
   
    5. Fig. 1C would be easier to understand if bar colors would be matched to the color scheme used in Fig. 1B.

**This is a nice idea but turned out to be confusing with so many colors. To make things simpler we have adopted a more neutral color scheme throughout the figure.**
   
    6. Fig. 2 panel D is missing its label "D".

**Thank you – we have done this.**
   
    Reviewer #2: (No Response)
   
    Reviewer #3: - In Fig 1D it would be nice to see if there was any killing in a carrier-solvent only control equivalent to the high-HHQ conditions.  From the methods, I actually could not discover what the carrier solvent was, but I am guessing from some of the figure makings that it was DMSO?  I note that AQs like PQS and HHQ are very poorly soluble in water (and DMSO) and I wonder if the authors have considered caveats that might be inherent in the exogenous addition killing experiments due to the potential for delivery of uneven and locally high concentrations of AQs resulting from compound aggregation?

**Thank you for highlighting this. In every experiment our negative control is indeed carrier (DMSO) alone at the same concentration as used for the AQs. We have modified our methods section to clarify this important point.**
   
    - Since multiple populations are generally being compared back to the same control population in most of the analysis for this manuscript, it would be more appropriate to perform ANOVA followed by post-hoc testing rather than multiple t-tests.

**Again, thank you for catching a point of confusion: in fact, we are not comparing back to the same control but rather use independent controls for each experiment that we measure at the same time as the experimental condition. Again, we have edited the methods to clarify this point.**
   
    - In using propidium iodide to stain for mammalian cell killing in the surface attached killing assay, is there a risk that unintended staining of attached bacterial extracellular matrix could be confounding?  I note the statement in the methods: “Bacterial co-culture with monocytes resulted in substantial monocyte lysis (see Figures 4A) and subsequent diffusion of the nuclear PI stain”.  Could the diffuse staining also be due to the presence of extracellular bacterial DNA?

**To address this concern we only measure the changes in PI staining within the monocytes. We have edited the methods to clarify this.**
   
    - The sentence beginning on Line 61 does not make sense and appears likely to be the result of a simple editing error.

**Thank you – we have fixed this.**

    --------------------
   
    PLOS authors have the option to publish the peer review history of their article (<a href="<https://journals.plos.org/plospathogens/s/editorial-and-peer-review-process#loc-peer-review-history>" target="_blank">what does this mean?</a>). If published, this will include your full peer review and any attached files.<br><br>
   
    If you choose “no”, your identity will remain anonymous but your review may still be made public.<br><br>
   
    <b>Do you want your identity to be public for this peer review?</b> For information about this choice, including consent withdrawal, please see our <a href="<https://www.plos.org/privacy-policy>" target="_blank">Privacy Policy</a>.
   
    Reviewer #1: No
   
    Reviewer #2: No
   
    Reviewer #3: No
   
    __________________________________________________
    In compliance with data protection regulations, you may request that we remove your personal registration details at any time.  (Use the following URL: <https://www.editorialmanager.com/ppathogens/login.asp?a=r>). Please contact the publication office if you have any questions.
