# Supplementary material for: Cytotoxic alkyl-quinolones mediate surface-induced virulence in Pseudomonas aeruginosa
Source: PLoS Pathog. 2020 Sep 14;16(9):e1008867. doi: 10.1371/journal.ppat.1008867 (PMC7515202; doi:10.1371/journal.ppat.1008867)
Supplement: Attachment — Submitted filename: Response to Reviewers.docx [file ppat.1008867.s020.docx]

Reviewer's Responses to Questions

**Part I - Summary**
Please use this section to discuss strengths/weaknesses of study, novelty/significance, general execution and scholarship.

Reviewer #1: This is a well-written and technically sound manuscript that describes a mechanism of surface-dependent killing by P. aeruginosa. Challenging, however, is the situational context to which the findings apply and might translate to actual mechanisms of virulence. The authors propose that attachment of Pa to glass activates surface dependent killing and all of the reported findings are in the context of a glass surface. How is attachment to a glass surface relevant? Does attachment to plastic trigger the same response? What is the relevant in vivo attachment surface? It would have to be a host surface, either cellular or matrix. Does attachment to host surfaces trigger the same response?

**This is an important point and we thank the reviewer for bringing it up. We previously showed that activation of virulence in response to surface attachment is independent of the type of surface to which *P. aeruginosa* is attached (Siryaporn et al. 2014). Given the importance of this result and its implications for the *in vivo* relevance of this phenomenon, in our current work we have expanded on these results with a new Figure S1. We now show a direct comparison of surface-dependent virulence following attachment to glass, plastic, agar, PDMS, and agarose with various stiffnesses within the stiffness regime of biological tissues (5 kPa -300 kPa).**

**Furthermore, we used our fluorescent AQ biosensor to directly test whether attachment of *P. aeruginosa* to a biological surface, a monolayer of lung epithelial cells, influences AQ levels. In the revised manuscript, we demonstrate that attachment to a monolayer of A549 human lung epithelial cells stimulates a significant increase in AQ production.**

Whereas the MOI for the Dictyo experiments was 500-1000, the monocyte experiments used an MOI of 0.02 – 0.007. It is hard to know whether an MOI of 500-1000 is physiologically relevant but there is some concern that killing ~90% of the Dictyo within 60 minutes (Fig. 1F) is an artefact of the MOI and in doing obscures more relevant mechanisms that function at a much lower (and possibly more relevant) MOI, such as the T3SS which can also contributes killing.

The cited study from the Iglewski group used also strain PA14 and THP-1 monocytes and observed nearly 100% lysis at 6 hours using an 0.1-1 MOI. Fig. 1F shows only 25% killing after 18 hrs. Does attachment to glass suppress T3SS-dependent killing?

**First of all, we thank the reviewer for bringing to our attention an important typo that led to this confusion. Specifically, we had labeled the MOIs for all of the monocyte experiments backwards: they were 50:1 – 150:1, as opposed to 1:50-1:150. In other words, we actually used similar MOIs for our Dicty and monocyte experiments.**

**Nevertheless, recognizing the importance of exploring the role of MOI in greater depth, we have performed additional experiments. Specifically, we show that while the MOI of 1000:1 is used for most of the reported experiments with *D. discoideum*, rapid killing still occurs even at substantially lower MOIs. For example, Supplemental Movie 2 shows the response of *D. discoideum* to surface attached *P. aeruginosa* at a MOI of 50:1. To emphasize this point, we have now included a new supplemental figure (S3) that shows the killing of *D. discoideum* treated with wild type and *pqsA* mutant PA14 at 5 hours of incubation at MOIs of 500:1, 250:1, 50:1, 25:1. Under no conditions do we observe any significant killing of *D. discoideum* by the *pqsA* mutant, so it is not likely that other virulence factors are acting at lower MOI. Wild type bacteria exhibit high virulence after 5 hr at a MOI of 50:1, but are no longer virulent at an MOI of 25 to 1. These relatively high MOIs (>50:1) are likely necessary for infection of *D. discoideum* given its ability to rapidly phagocytose a bacterial lawn as demonstrated in Supplemental Movie 1 and now Figure S3.**

**We believe that dynamic changes in MOI during the course of the experiments can also explain why the Iglewski group observed complete PA14 killing of monocytes. In the Iglewski paper, they report the MOI as 0.1-1 as those were the ratios at the time of the start of the experiment. However, since they did nothing to suppress bacterial growth and the bacteria grow exponentially far faster than the monocytes, at 6 h (the time when killing was measured), the PA14:THP-1 ratio was approximately 750:1 as confirmed by cfu counting. To avoid such dramatic changes in MOI during the course of the experiment, our setup always includes a sub-MIC level of a bacteriostatic antibiotic to limit bacterial growth. To show both that this MOI difference can indeed explain the difference between their findings and ours and that the presence of the sub-MIC antibiotic does not itself disrupt virulence, we confirmed in our setup that at approximately 500:1 MOI we observed nearly complete monocyte killing after 5 h:**

Reviewer #2: The authors were very responsive to my comments. The revised manuscript now contains (again?) additional data on OMVs, demonstrating OMV trafficked AQs display enhanced cytotoxicity towards host cells, but do not enhance their efficacy as autoinducers. These additional experiments are also well executed and controlled. In conclusion, the revised manuscript is well-written and -presented, with clear and well controlled experiments that justify the conclusions drawn.

Reviewer #3: In this study Vrla et al. aimed to assess and identify the possible role of AQs produced by P. aeruginosa during infection, and they investigated the role of AQs production in response to the surface attachment. To do so, they used Dictyostelium and mammalian monocytes as infection/cytotoxicity model systems.

The authors showed a significant decrease in Dictyostelium cell death in pqsA and pqsR mutants, leading them to the finding that HHQ and PQS are responsible for both mammalian and Dictyostelium cells. However, HHQ is sufficient for cytotoxicity against Dictyostelium. The authors developed several bioreporters that confirm higher levels of expression of AQs in surface-attached cells compared to planktonic cells.

One of the central and exciting findings is the increased cytotoxicity of intact OMVs compared to the lysed OMVs and the imaging methods used.

I found the regulatory role of small RNA Lrs1 one of the main findings of this manuscript that connects two main QS regulatory systems in P. aeruginosa. However, this finding is a bit lost. Maybe more discussion on complicated QS system and it’s role in virulence during chronic infections and biofilms of P. aeruginosa can add more emphasis to this finding.

**Thank you for mentioning this point. We were previously criticized for being too speculative about potential distinct roles for QS in different contexts so we are reluctant to be too specific about what might be going on. Nevertheless, we agree with the spirit of the reviewer’s comments and have thus added additional text to the discussion to highlight the implications for our Lrs1 findings for QS interconnectivity.**

**Part II – Major Issues: Key Experiments Required for Acceptance**
Please use this section to detail the key new experiments or modifications of existing experiments that should be absolutely required to validate study conclusions.

Generally, there should be no more than 3 such required experiments or major modifications for a "Major Revision" recommendation. If more than 3 experiments are necessary to validate the study conclusions, then you are encouraged to recommend "Reject".

Reviewer #1: (No Response)

Reviewer #2: N/A

Reviewer #3: (No Response)

**Part III – Minor Issues: Editorial and Data Presentation Modifications**
Please use this section for editorial suggestions as well as relatively minor modifications of existing data that would enhance clarity.

Reviewer #1: (No Response)

Reviewer #2: N/A

Reviewer #3: line 184: Not sure if the surface attachment leads to AQs level beyond reported previously, couldn't it be that when other virulence factors are available, small concentrations of HHQ is enough for the levels of cytotoxicity seen by whole cells?

**Thank you for mentioning this point. We agree, and believe that the situation may not be as simple as just these two possibilities we originally described and have edited the text to reflect this.**

Figure 2 A and D, can be moved to the supplementary documents.

**While we appreciate the reviewer’s suggestion, we respectfully believe that the quantification of the biosensor to multiple AQs using a plate reader (Fig 2A) and the standard curve for HHQ (Fig 2C) using imaging are key results and thus prefer to leave them in the main text. We also now measure AQ production by WT bacteria attached to a A549 monolayer, so it’s helpful to see a standard curve for PQS as well as HHQ.**

Figure 4. E can be moved to the supplementary documents, this will allow more resolution and emphasis on sections C and G.

**Since the importance of the images in C is to illustrate the general trend in fluorescence levels (as opposed to single cell resolution), we would prefer to keep these data in the main figure.**

Despite the efforts on OMV isolation from planktonic cultures, it would be a valuable addition in follow up studies to isolate the OMVs from biofilms or surface attached cells.

**Thank you for this very interesting idea. Since almost all reports of OMV isolation use liquid cultures, it would be interested to see if OMVs from surface associated bacteria exhibit unique features. We hope to pursue this idea in the future, but believe it is beyond the scope of the current paper.**

PLOS authors have the option to publish the peer review history of their article ([what does this mean?](https://journals.plos.org/plospathogens/s/editorial-and-peer-review-process#loc-peer-review-history)). If published, this will include your full peer review and any attached files.


If you choose “no”, your identity will remain anonymous but your review may still be made public.


**Do you want your identity to be public for this peer review?** For information about this choice, including consent withdrawal, please see our [Privacy Policy](https://www.plos.org/privacy-policy).

Reviewer #1: No

Reviewer #2: No

Reviewer #3: No
